# Reconstruction of Oromandibular Defects With Radial Forearm Free Flaps and Fibular Free Flaps: One-Year Experience From Hospital Selayang and the National Cancer Institute, Malaysia

**DOI:** 10.7759/cureus.92146

**Published:** 2025-09-12

**Authors:** Mohammad Azrul Abdul Rashid, Syahril Rizal Arsad, Rashdeen Fazwi Muhammad Nawawi

**Affiliations:** 1 Hand and Microsurgery Unit, Department of Orthopedics, Hospital Selayang, Kuala Lumpur, MYS

**Keywords:** donor-site morbidity, fibular free flap, free tissue transfer, head and neck reconstruction, oromandibular reconstruction, radial forearm free flap

## Abstract

Background

Oromandibular defects are complex to reconstruct, often requiring free tissue transfer. Radial forearm free flaps (RFFFs) and vascularized fibular free flaps (VFFFs) are among the most established techniques. This study aimed to evaluate short-term outcomes, complications, and donor-site morbidity of RFFFs and VFFFs in oromandibular reconstruction over a one-year period at Hospital Selayang and the National Cancer Institute, Malaysia.

Methodology

A retrospective review was conducted of patients who underwent oromandibular reconstruction with RFFFs or VFFFs between July 2022 and July 2023. Outcomes assessed included flap survival, donor-site morbidity, and patient satisfaction.

Results

A total of 16 patients were included, of whom 10 received an RFFF and six received a VFFF. All RFFF cases achieved 100% early flap survival, while one VFFF case (13%) failed and required removal. Donor-site morbidity in the RFFF group included superficial radial nerve sensory deficits (70%), cold intolerance (40%), and dissatisfaction with scarring (40%). In the VFFF group, no foot drop, nerve injury, or ankle instability occurred; however, leg swelling (40%) and cold intolerance (40%) were reported.

Conclusions

Both RFFF and VFFF are effective for oromandibular reconstruction. RFFF is more suitable for soft tissue defects, while VFFF remains the preferred option for osseous reconstruction. To maximize outcomes, it is necessary to individually select flaps based on defect characteristics and patient factors.

## Introduction

Oromandibular defects, defined as composite defects involving the oral cavity and mandible, result from ablative surgery for malignancies, trauma, or osteoradionecrosis and present a significant reconstructive challenge [1-3]. Reconstruction of oromandibular defects is critical to restore aesthetic appearance and re-establish essential functions such as speech articulation, mastication, swallowing, and airway patency [4]. The complex anatomy and the multifunctional role of the head and neck region make reconstruction particularly challenging, with the goal of achieving both structural integrity and functional rehabilitation.

Traditional local and regional flaps often fall short for extensive or composite defects, limited by reach, bulk, and lack of structural support [5]. The advent of microvascular free tissue transfer has revolutionized head and neck reconstruction, allowing tailored, one-stage repair with improved outcomes [6,7].

The radial forearm free flap (RFFF) and the vascularized fibular free flap (VFFF) are two workhorse flaps for oromandibular reconstruction [1,3,6]. The RFFF, known for its thin, pliable skin and reliable vascular pedicle, is particularly useful for soft tissue reconstruction [2,4]. The VFFF, with its robust bone stock, is ideal for reconstructing segmental mandibular defects, often requiring osteotomies for contouring [6].

This study presents a retrospective observational analysis of our one-year experience at the Hand and Microsurgery Unit, Hospital Selayang and the National Cancer Institute of Malaysia, focusing on the surgical outcomes and donor-site complications of oromandibular defect reconstruction using the RFFF and VFFF. We aim to evaluate the early graft survival and donor-site morbidities and complications associated with these reconstructive techniques in our patient population. By sharing our outcome and findings, we hope to contribute to the existing body of knowledge and refine our reconstructive strategies for improved patient outcomes.

## Materials and methods

Study design

This was a retrospective observational study conducted between July 2022 and July 2023 at the Maxillofacial Department, National Cancer Institute, Malaysia.

Patient selection

Patients with oral cancer who underwent oromandibular reconstruction with microvascular free flaps were included. The inclusion criteria were histologically confirmed oral cancer requiring segmental or composite resection with immediate reconstruction, and reconstruction performed using either an RFFF or a VFFF. Patients were included if the extent of the defect involved significant soft tissue loss of the oral cavity (requiring pliable thin coverage with RFFF) or segmental mandibular bone loss exceeding 4 cm, or composite defects involving both bone and soft tissue (necessitating VFFF). Only patients with defects severe enough to preclude primary closure or local flap reconstruction were considered eligible.

We excluded patients with recurrent oral cancer, previous head and neck free flap reconstruction, or inadequate follow-up data. Patients with significant peripheral vascular disease, poor recipient vessel quality, or contraindications to microvascular surgery (such as severe cardiopulmonary comorbidities) were also excluded. Individuals with defects small enough to be managed with primary closure, local flaps, or regional pedicled flaps were also not considered for inclusion.

Preoperative evaluation

All patients underwent standardized preoperative assessment, including routine oncologic and anesthetic work-up. For VFFF candidates, CT angiography of the lower limbs was performed to assess vascular anatomy. For RFFF candidates, CT angiography of the upper limbs and an Allen test were conducted to confirm the adequacy of ulnar artery circulation, as well as to avoid any vascular insufficiency after RFFF harvesting.

Surgical technique

For RFFF, the non-dominant forearm was preferred and harvested using the standard technique. VFFF was harvested using the lateral approach described by Gilbert [8]. In both groups, arterial anastomosis was performed end-to-end with the facial artery. Venous drainage was via the thyrolinguofacial trunk or tributaries, with secondary outflow through the external jugular vein. All procedures were performed by the same surgical team. Postoperatively, flaps were monitored by color change, temperature, and the pin-prick method every two hours for the first 48 hours and then less frequently.

Outcome measures

The primary outcome was early flap survival, defined as a viable, functioning free flap without revision or necrosis at 30 days postoperatively. Early flap failure was defined as total flap loss within 30 days, requiring debridement and removal [9].

Secondary outcomes comprised donor-site complications, patient-reported satisfaction, and wound-healing status. Donor-site morbidity for both the RFFF and VFFF was assessed for skin graft uptake and wound healing (classified as good when epithelialization was complete without breakdown by three weeks and delayed when breakdown or persistent exudate extended beyond three weeks or when >10% graft loss occurred) [10]. Both groups of patients were also evaluated for cold intolerance, defined as patient-reported discomfort, pain, numbness, or color change of the donor limb with cold exposure.

The RFFF donor site was specifically assessed for superficial radial nerve sensory deficit (presence of numbness, paresthesia, or reduced light-touch/two-point discrimination in the superficial radial nerve distribution reported by the patient or noted on follow-up examination), aesthetic outcome (patient-reported appearance rated on a five-point Likert scale and dichotomized as satisfactory versus poor), and hand function (patient-reported ability to perform daily activities, grip-related tasks, and absence of limiting stiffness; dichotomized as good versus poor).

Patients who underwent VFFF were examined for foot drop, defined as ankle dorsiflexion strength <4/5 on the Medical Research Council scale or a need for ankle-foot orthosis, ankle pain, or instability, defined as persistent symptoms that limit ambulation or require bracing [11]. Donor-leg swelling was defined as clinically evident edema persisting beyond two weeks or requiring compression therapy [11].

All outcomes were abstracted from medical records and follow-up notes and supplemented by telephone interviews to ensure completeness and consistency.

Statistical analysis

Data were analyzed using descriptive statistics. Continuous variables were reported as means with ranges, while categorical variables were expressed as frequencies and percentages. No comparative statistical tests were applied due to the small sample size.

## Results

A total of 16 patients underwent oromandibular reconstruction during the study period. Overall, 10 patients underwent reconstruction with RFFF, while six patients underwent VFFF. The mean age was 52 years (range = 20-67), with a male-to-female ratio of 9:7 (Table 1).

**Table 1:** Patient demographics. RFFF: radial forearm free flap; VFFF: vascularized fibular free flap

Sex	RFFF (n = 10)	VFFF (n = 6)	Total no (n = 16)
Male	5 (50%)	4 (67%)	9 (56%)
Female	5 (50%)	2 (33%)	7 (44%)
Mean age	45 (20–67)	50 (20–67)	52 (20–67)

A total of eight patients who underwent RFFF had lesions on the tongue and floor of the mouth, and two patients had lesions on the buccal mucosa. All six patients who underwent VFFF had a lesion in the mandible that required mandibular reconstruction (Table 2).

**Table 2:** Pathology characteristics. RFFF: radial forearm free flap; VFFF: vascularized fibular free flap

Site of the lesion	RFFF (n = 10)	VFFF (n = 6)
Tongue/Floor of the mouth	8 (80%)	0 (0%)
Buccal mucosa	2 (20%)	0 (0%)
Mandible	0 (0%)	6 (100%)

All patients in the RFFF group (n = 10) achieved 100% early flap survival. In the VFFF group, five out of six patients (87%) had successful early flap survival, while one (13%) patient experienced early flap failure, requiring flap debridement (Table 3). The one patient with early flap failure later succumbed due to oncological complications and was excluded from the donor-site morbidity evaluation.

**Table 3:** Early flap survival. RFFF: radial forearm free flap; VFFF: vascularized fibular free flap

Variable	RFFF (n = 10)	VFFF (n = 6)
Early flap survival	10 (100%)	5 (83%)
Early flap failure	0 (0%)	1 (17%)

In the RFFF group, eight patients demonstrated good split-thickness skin graft uptake with satisfactory wound healing, while two experienced delayed healing; none required regrafting. Superficial radial nerve sensory deficits were reported by seven patients, either partial or complete, whereas three had no sensory disturbance. Six (60%) patients were satisfied with the donor-site appearance, while four (40%) patients expressed dissatisfaction, primarily due to hypertrophic or keloid scarring. Hand function remained preserved in nine patients, with only one patient reporting stiffness that impaired fine motor activity. Cold intolerance of the donor limb was observed in four patients, while the remaining six reported no such symptoms (Table 4).

**Table 4:** Donor-site morbidities and complications of RFFF. RFFF: radial forearm free flap

Morbidities/Complications	Number of patients	Number of patients
Skin graft uptake and wound healing	Good – 8 (80%)	Delayed – 2 (20%)
Superficial radial nerve sensory deficit	No deficit – 3 (30%)	Partial or complete deficit – 7 (70%)
Donor-site aesthetic result	Satisfactory – 6 (60%)	Poor – 4 (40%)
Function of the donor hand	Good – 9 (90%)	Poor – 1 (10%)
Cold intolerance of the donor limb	Absent – 6 (60%)	Present – 4 (40%)

In the VFFF group, four patients demonstrated good skin graft uptake with uneventful healing, while one experienced delayed wound healing that did not require regrafting. None of the patients developed neurological or functional complications. In particular, there were no cases of common peroneal nerve injury, foot drop, ankle pain, or ankle instability. Donor-leg swelling was reported by two patients, while the remaining three experienced no swelling. Cold intolerance of the donor limb was reported in two patients, with three patients denying such symptoms (Table 5).

**Table 5:** Donor-site morbidities and complications of VFFF. VFFF: vascularized fibular free flap

Morbidities/Complications	Number of patients	Number of patients
Skin graft uptake and wound healing	Good – 4 (80%)	Delayed – 1 (20%)
Foot drop	Absent – 5 (100%)	Present - 0
Ankle pain and instability	Absent – 5 (100%)	Present - 0
Leg swelling	Absent – 3 (60%)	Present – 2 (40%)
Cold intolerance of the donor limb	Absent – 3 (60%)	Present – 2 (40%)

## Discussion

This study presents one year of experience in oromandibular reconstruction using the RFFF and the VFFF at Hospital Selayang and the National Cancer Institute, Malaysia. Our results demonstrate a high overall flap survival rate, with all patients in the RFFF group achieving 100% early flap survival, while the VFFF group showed an 87% success rate, with one early flap failure requiring debridement. However, the patient with flap failure succumbed due to oncological complications.

The flap survival rate in our RFFF cohort is consistent with international literature, where success rates typically range between 95% and 100% due to its reliable vascular anatomy and ease of harvest [12-14]. The slightly lower survival rate in the VFFF group reflects the increased complexity associated with harvesting a composite flap that includes both bone and soft tissue. Despite this, the majority (87%) of patients who underwent VFFF reconstruction had successful early flap outcomes, confirming its effectiveness for mandibular reconstruction, particularly in cases involving segmental bone defects [12-15].

In terms of donor-site morbidity, RFFF demonstrated some expected complications. While 80% of patients had good skin graft uptake, 20% experienced delayed wound healing due to poor graft take, though none required re-grafting. A notable complication was the high incidence of superficial radial nerve sensory deficits, reported in 70% of patients, which is consistent with previous studies that cite sensory loss as a common issue due to the proximity of the nerve during flap harvest [16]. Despite this, 90% of patients reported no functional deficits of the donor hand, and only one patient described hand stiffness. Cold intolerance was reported in 40% of RFFF cases, an often underreported but significant postoperative symptom.

Aesthetic satisfaction with the RFFF donor site varied, with 60% of patients expressing satisfaction, while the remaining 40% were dissatisfied primarily due to visible forearm scarring. This highlights the importance of preoperative counselling and potential consideration of flap design techniques to minimize aesthetic morbidity [16-19].

For the VFFF group, donor-site morbidity was generally low [20]. Most patients (80%) had good skin graft healing at the lower leg, with no cases requiring re-grafting. Importantly, there were no reports of common peroneal nerve injury, foot drop, ankle instability, or pain, complications that are frequently considered when selecting the fibula as a donor site [21]. However, leg swelling was reported in 40% of VFFF patients, and cold intolerance was present in 40%, indicating that even in the absence of major complications, patients may still experience discomfort or functional limitations that impact quality of life [14,15].

Overall, our findings affirm that both RFFF and VFFF are reliable options for oromandibular reconstruction, with flap selection largely determined by defect characteristics. RFFF remains a preferred choice for soft tissue-only defects due to its thin, pliable nature and short harvest time [19,22]. VFFF, with its ability to provide vascularized bone, remains the gold standard for large mandibular reconstruction despite the more technically demanding harvest and slightly higher donor-site burden [6,7].

Limitations

This study is limited by its small sample size and single-center retrospective design, which may affect the generalizability of the findings. Additionally, the follow-up period was limited to the early postoperative phase. Further, long-term functional outcomes (e.g., speech, swallowing, masticatory function) and patient-reported quality of life were not assessed. The subjective nature of some complications, such as cold intolerance and aesthetic satisfaction, also introduces variability in reporting.

Recommendations and future directions

Further studies involving larger cohorts and longer follow-up periods are needed to better evaluate functional recovery and patient-centered outcomes. Future research should incorporate validated outcome measures and quality of life assessments to more accurately reflect the impact of flap choice on postoperative recovery and satisfaction. A prospective or multicenter study design would also strengthen the evidence base and guide flap selection protocols in various clinical contexts.

## Conclusions

Both the RFFF and the VFFF remain reliable options for oromandibular reconstruction. RFFF demonstrated excellent flap survival with minimal functional impairment, making it suitable for soft tissue defects, although donor-site morbidity such as sensory loss and aesthetic dissatisfaction should be anticipated. VFFF showed slightly lower flap survival but remains the preferred option for mandibular and composite defects, with generally low rates of major donor-site complications; minor issues such as swelling and cold intolerance were observed without significant functional limitation. Flap selection should be individualized based on defect characteristics, patient factors, and anticipated donor-site outcomes. Careful preoperative evaluation, patient counseling, and a multidisciplinary approach are critical to optimize functional recovery and patient satisfaction. Larger studies with longer follow-up are needed to clarify long-term functional outcomes and quality of life measures for both techniques.
